# Control of T lymphocyte fate decisions by PI3K signaling

**DOI:** 10.12688/f1000research.26928.1

**Published:** 2020-09-25

**Authors:** Benjamin Murter, Lawrence P. Kane

**Affiliations:** 1 Department of Immunology, University of Pittsburgh School of Medicine, Pittsburgh, PA, 15261, USA

**Keywords:** Lymphocyte activation, signalling, lipid kinases, regulatory T cells

## Abstract

Virtually all aspects of T and B lymphocyte development, homeostasis, activation, and effector function are impacted by the interaction of their clonally distributed antigen receptors with antigens encountered in their respective environments. Antigen receptors mediate their effects by modulating intracellular signaling pathways that ultimately impinge on the cytoskeleton, bioenergetic pathways, transcription, and translation. Although these signaling pathways are rather well described at this point, especially those steps that are most receptor-proximal, how such pathways contribute to more quantitative aspects of lymphocyte function is still being elucidated. One of the signaling pathways that appears to be involved in this “tuning” process is controlled by the lipid kinase PI3K. Here we review recent key findings regarding both the triggering/enhancement of PI3K signals (via BCAP and ICOS) as well as their regulation (via PIK3IP1 and PHLPP) and how these signals integrate and determine cellular processes. Lymphocytes display tremendous functional plasticity, adjusting their metabolism and gene expression programs to specific conditions depending on their tissue of residence and the nature of the infectious threat to which they are responding. We give an overview of recent findings that have contributed to this model, with a focus on T cells, including what has been learned from patients with gain-of-function mutations in PI3K as well as lessons from cancer immunotherapy approaches.

## Antigen receptor signaling and PI3K

T and B cells are subject to a constant barrage of extracellular signals and cues, and the combined effects of these various signals decide the eventual functions and fate of these cells. These cellular cues, whether they are sensed through receptors for antigen (T cell receptor for antigen [TCR]), membrane immunoglobulin (mIg), cytokines, or other growth factors, are integrated in lymphocytes by signaling pathways that sustain and/or trigger the differentiation of these cells. The key elements of early signaling through the antigen receptors include tyrosine kinases of the Src and Syk families, phosphorylation of adaptor proteins, and recruitment and activation of PLC-γ ^1, 2^. Activated PLC-γ mediates the production of the critical second messengers diacylglycerol (DAG) and inositol tris-phosphate (IP _3_), which lead to activation of PKC/Ras and calcium-dependent signaling pathways, respectively.

**Figure 1.**
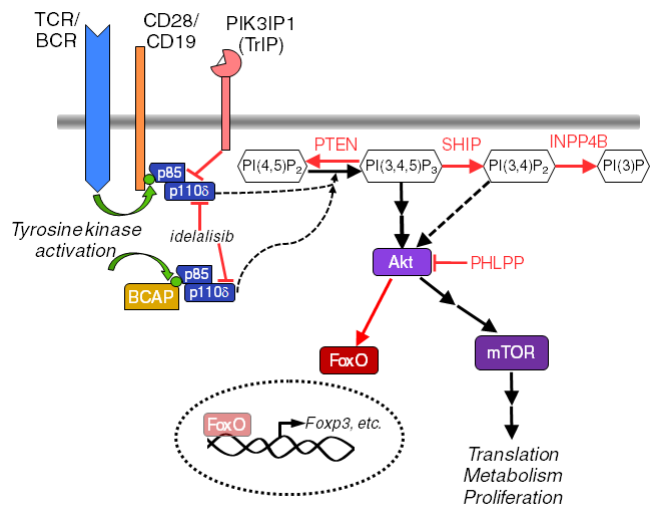
Major regulators and effectors of the phosphatidylinositol 3-kinase (PI3K) pathway in lymphocytes. Molecules and interactions discussed in the text are highlighted here. Activating events are indicated with black arrows. Inhibitory events are depicted with red lines or arrows. This is not an exhaustive model of the pathway. BCAP, B-cell adapter for PI3K; BCR, B cell receptor; INPP4B, inositol polyphosphate-4-phosphatase type II B; mTOR, mammalian target of rapamycin; PHLPP, PH domain and leucine-rich repeat protein phosphatase; PIK3IP1, PI3K interacting protein 1; PTEN, phosphatase and tensin homolog deleted on chromosome 10; SHIP, src homology 2 containing 5’-inositol phosphatase; TCR, T cell receptor.

The phosphatidylinositol 3-kinase (PI3K) signaling pathway, present in all eukaryotic cells, is another signaling pathway that acts as a key regulator of B and T cell fates. An overview of PI3K signaling pathways discussed in this review is shown in Figure 1.

There are three different classes of PI3K (I, II, and III) as well as various isoforms that comprise those classes. Here we will mainly address the class IA PI3K PI3Kδ, which is the isoform primarily activated by lymphocyte antigen receptors ^3, 4^. Class IA PI3Ks are heterodimeric kinases consisting of a regulatory subunit (p85α, p55α, p50α, p85β, or p55γ) encoded by three genes ( *Pik3r1*, *Pik3r2*, and *Pik3r3*) and a catalytic subunit (p110 α, β, or δ). Activation of the catalytic subunits downstream of receptor-tyrosine kinases (RTKs) leads to the catalytic conversion of the phospholipid PI(4,5)P _2 _to the membrane-bound second messenger phosphoinositide 3,4,5-triphosphate (PIP _3_) ^5^. PIP _3_ recruits a number of pleckstrin homology (PH) domain-containing proteins, including the serine/threonine (S/T) PIP _3_-dependent kinase 1 (PDK1), which in turn phosphorylates and activates the S/T kinases of the Akt (aka protein kinase B, PKB) family (there are three Akt family members in mammals, Akt1–3). Activated receptors recruit class IA PI3Ks via binding of one or more of their SH2 domains to phosphorylated tyrosines in such receptors. This binding appears to occur most efficiently at phosphorylated tyrosines within YXXM sequences ^6^.

In T cells, the best-defined receptor for PI3K recruitment is the co-stimulatory protein CD28, a transmembrane protein that binds to B7 family proteins expressed by antigen-presenting cells (APCs). This interaction is critical for the activation of naïve T cells; however, the relevance of PI3K binding to the co-stimulatory activity of CD28 has been controversial ^7^. This is, in part, because of the expanding understanding of the distinct and nonredundant role of ICOS (a CD28 family member) and its potent co-stimulation of the PI3K pathway in T cells ^8– 10^. Recent reports detailing how these family members promote such seemingly divergent T cell fates are discussed later.

In B cells, the recruitment of PI3K into the proximity of engaged antigen receptors mainly occurs through the transmembrane protein CD19, which functions as part of a co-receptor complex that includes complement receptor 1 (CD21) and the tetraspan protein CD81 ^11^. Coordination of this co-receptor signaling is performed in part by B-cell adapter for PI3K (BCAP), an adaptor protein capable of integrating multiple downstream signaling pathways ^12, 13^. This seems to be mediated by the ability of BCAP to associate with GRB2 and the p85 domain of the inactive form of PI3K (among other proteins) ^14, 15^. Despite its initial discovery in B cells and the absence of its expression in naïve T cells, BCAP is upregulated in T cells following activation, and we will highlight the recent discoveries involving T cells within the scope of this review ^16^. These studies have revealed that BCAP plays a key role in the clonal expansion of effector and memory CD8 ^+^ T cells and in the differentiation of Th1 and Th17 helper T cells ^17^. Together, these data suggest that BCAP upregulation in differentiated T cells serves to help assemble signaling complexes important for priming T cells for secondary responses to antigen. While these functions of BCAP are ascribed to its YXXM motifs and the recruitment and activation of PI3K, the presence of other protein–protein interaction domains within BCAP suggests that it has the ability to coordinate signaling through multiple pathways.

It should be noted that a variety of receptors, including antigen, co-stimulatory, cytokine, chemokine, Toll-like, and growth factor receptors, are all known to activate PI3K signaling, and lymphocytes may receive input from many of these receptors concurrently ^18^. This should be considered when interpreting the results of experiments to manipulate signaling through this pathway.

## Negative regulators of PI3K signaling in lymphocytes

As with any signaling pathway that controls cellular growth and survival, the PI3K pathway is also subject to negative regulation. The majority of PI3K signal regulation is performed by two lipid-phosphatases: phosphatase and tensin homolog deleted on chromosome 10 (PTEN) and src homology 2 containing 5’-inositol phosphatase (SHIP), which convert PIP _3_ to PI(4,5)P _2_ and PI(3,4)P _2_, respectively. There is an additional negative regulator of PIP _3_, known as INPP4B (inositol polyphosphate-4-phosphatase type II B), which acts on the 4’ phosphate, although this is less well understood and has not been studied in any detail in lymphocytes. Highlighting the importance of proper PI3K regulation, loss-of-function mutations in PTEN or SHIP have been linked to tumorigenesis ^19, 20^. Expression of PTEN appears to be particularly important, as homozygous loss of PTEN leads to early lethality, by embryonic day 7.5 in mice ^21^. Interestingly, heterozygous loss of PTEN also leads to a distinct type of pathology, in the form of a group of hereditary disorders known as PTEN hamartoma tumor syndrome (PHTS). These patients display immune dysregulation due to enhanced PI3K/Akt signaling ^22^, which was postulated to lead to the disruption of T cell development.

Further study of PHTS patients led to the discovery of PH domain and leucine-rich repeat protein phosphatase (PHLPP) as a regulatory phosphatase downstream of PI3K and PTEN with a unique role in regulatory T cells (Tregs) ^23^. PHLPP isoforms (PHLPP1 and PHLPP2) were shown to regulate the activity of the PI3K effector Akt through the dephosphorylation of S473 in Akt ^24^. The expression of PHLPP has been shown to be particularly elevated in Treg cells, where it acts to preserve their suppressive phenotype in coordination with—and/or as compensation for—PTEN. More recently, a transmembrane inhibitor of PI3K, PI3K interacting protein 1 (PIK3IP1) also known as transmembrane inhibitor of PI3K (TrIP), has been described ^25, 26^. The maintenance of PI3K pathway homeostasis by these negative regulators, and their influence on lymphocyte differentiation, will be discussed further below.

## Effects of PI3K signaling on lymphocyte differentiation

PI3K signaling is instrumental in promoting the inflammatory activity of T cells through phosphorylation of the FOXO transcription factors, leading to their nuclear exclusion and subsequent downregulation of cellular adhesion markers (e.g. CD62L and CCR7). This promotes trafficking of T cells out of secondary lymphoid tissues and into sites of inflammation. While nuclear exclusion of FOXO helps drive the inflammatory response, re-expression of FOXO1 is indispensable for CD8 ^+^ memory T cell maintenance and self-renewal ^27^. Recent evidence demonstrating the upregulation of BCAP in CD8 ^+^ memory T cells contributes to our understanding of how these cells are primed to respond upon subsequent antigen encounter ^28^.

PI3K signaling and some of its major downstream effectors such as mTOR and FOXO are necessary for the generation of helper T cell effectors (Th1, Th2, Th17), while conversely these factors are thought to be detrimental to the formation of Tregs ^29– 32^. Building on this seminal work, numerous studies have investigated the context-specific roles of these signals in both mouse models and human disease. Below we will discuss the requirement of PI3Kδ signaling in Tregs and speculate on how these cells have evolved to fine-tune PI3K/Akt signaling, which can be detrimental to their function. We will also examine recent discoveries in activated PI3Kδ syndrome (APDS) and how PI3K dysregulation disproportionally affects Tfh cells and the subsequent B cell response. The PI3K pathway is highly regulated through downstream negative regulation, i.e. turnover of PIP _3_ or de-phosphorylation of downstream targets, e.g. Akt. In this review, we will discuss recent studies that demonstrate the role of a more novel membrane-bound inhibitor of PI3K, PIK3IP1, which operates upstream of any previously described regulators of this pathway. The implications of this novel membrane-bound regulator within the context of cancers as well as other studies regarding the use of PI3K inhibitors and their immunotherapeutic applications are discussed, as are some of the lessons that have been learned so far from these studies.

## Complex roles of PI3K and its effectors in regulatory T cell biology

The role of PI3K signaling in the development, maintenance, and function of Tregs is not short on controversy. The strongest evidence that PI3K signaling limits Treg development comes from studies using the p110δ ^D910A^ mouse model, in which p110δ is inactivated by a point mutation ^33^. Importantly, these mice avoid possible confounding effects of a total knockout of all p110δ protein, an approach that results in secondary effects of reduced stability of associated PI3K regulatory subunits ^34^. Thus, thymic-derived Tregs (also known as natural Tregs, nTregs) are found in much greater proportions in p110δ ^D910A^ mice ^35^, at least in the thymus itself. By contrast, the spleen and lymph nodes of p110δ ^D910A^ mice contain reduced numbers of Tregs, which are also less suppressive *in vitro* and fail to protect against experimental colitis *in vivo* ^35^ *.* Tregs can also be generated outside the thymus from naïve CD4 ^+^ T cells; these cells are termed “induced Tregs” or iTregs. This process can be modeled *in vitro* and indeed is more efficient under conditions of limiting PI3K/Akt/mTOR activity ^30, 36, 37^.

Further highlighting a role for PI3K in limiting Treg development, inhibition or knockout of the mechanistic target of rapamycin (mTOR) complexes, which are downstream effectors of PI3K, results in *de novo* expression of Foxp3 in naïve T cells following initial activation ^31^. These studies raise a paradox regarding the role of PI3K in Treg generation and suppressive function. Thus, partial inhibition of PI3K or its downstream effectors (Akt, mTOR) results in the promotion of Foxp3 expression upon stimulation, yet the complete loss of PI3K signaling results in inefficient Treg generation and function ^38^. These seemingly contradictory effects of PI3K modulation may be explained mechanistically by understanding that the downstream biology depends on some combination of context, timing, and overall magnitude of PI3K signaling. This requirement for tight control also suggests the potential for future discovery of additional endogenous or therapeutic regulators that fine-tune PI3K within Tregs.

One potential mechanism by which Tregs uniquely integrate these seemingly competing PI3K signaling requirements is via the expression of PHLPP. As described above, Tregs have elevated expression of PHLPP compared to other T cell populations. Among other possible mechanisms, negative regulation of PI3K/Akt signaling by PHLPP could serve to prevent Akt-mediated FOXO nuclear exclusion and thereby preserve Foxp3-dependent transcriptional programs in the presence of PI3K signaling. This and other possible mechanisms, such as the influence of IL-2 signaling on the immunometabolism of Tregs, have been explored in greater detail by others ^32, 39^. Further support for the importance of tight PI3K signal regulation in Tregs comes from studies with selective PI3K inhibitors like the p110δ inhibitor idelalisib, which demonstrated preferential effects on Tregs in chronic lymphocytic leukemia (CLL) patients ^40^. Overall, study of the influence of PI3K on Treg biology has revealed the uniquely sensitive nature of these cells to either the absence or the excessive activation of this pathway, especially in comparison to other T cell types. Clearly, further study of the relationship between PI3K and Treg biology is warranted.

## Lessons from activated PI3Kδ syndrome

Much of our understanding of PI3K signaling has come from a variety of reductionist approaches using pharmacological inhibition, knockdown, and knockout of different PI3K subunits. A number of recent studies evaluating the effects of overactive PI3K signaling in APDS patients have helped reveal additional aspects of PI3K function in immunity and beyond ^41– 43^. Clinical presentation in this disease can be complex and variable; in one study of 53 patients, the most common findings were increased respiratory tract infections (98%), lymphoid hyperplasia (~60%), herpesvirus infections (49%), and autoimmunity (42%) ^44^. Facilitating the understanding of APDS, a mouse model of active PI3K (Pik3cd ^E1020K/+^) reproduces many aspects of this disease ^45– 49^. Clinical and cellular aspects of APDS have been reviewed extensively elsewhere ^50– 52^, so we will touch on a few highlights here.

APDS can result from mutations in either *PIK3R1* (p85 subunit) or *PIK3CD* (p110δ subunit), which ultimately leads to increased activity of PI3Kδ. Mechanistically, these mutations function either by disrupting the inhibitory contacts of p110 with p85 or by increasing the affinity of p110δ for the plasma membrane, promoting its interaction with PIP _2_^53^. Consistent with the known functions of PI3K signaling, APDS mutations result in enhanced activation of the Akt and mTOR kinase pathways and decreased activity of the FOXO1 transcription factor in T cells ^50^. These changes are associated with increased T cell effector function and enhanced migration out of secondary lymphoid organs. It was also recently shown that APDS mutations result in enhanced cytokine production by effector CD4 ^+^ helper T cells ^50, 54^. However, consistent with a role for PI3K in limiting Treg development and function (discussed above), *in vitro* differentiation of naïve CD4 ^+^ T cells into inducible Tregs was less efficient with APDS T cells ^50^.

The phenotype of T cells in mice or humans with activating mutations in PI3K presents a conundrum in that these mutations increase the sensitivity of T cells to activation while also resulting in immunodeficiency. As discussed below, chronic PI3K activity may actually promote T cell exhaustion and/or senescence, thereby predisposing affected individuals to chronic infection (see also 42, 50, 51). Most APDS patients present with an increase in circulating transitional B cells, as well as reduced numbers of class-switched B cells. In addition to clear B cell-intrinsic effects of APDS-associated mutations ^45– 47, 55^, the defects in humoral immunity could also be due in part to the elevated numbers of Tfh cells and disorganized germinal centers found within the secondary lymphoid organs of APDS patients ^56^. Within the germinal center, the signals that drive the Tfh program also initiate the expression of ICOS and migration towards the T-B border zone. ICOS expression is necessary for the formation of Tfh cells and cannot be compensated for by CD28, perhaps because ICOS is an even more potent activator of PI3K than is CD28 ^57^.

Along with high levels of ICOS, Tfh cells also express high levels of PD-1, a known inhibitor of CD28-mediated signaling ^58^. Therefore, ICOS expression coupled with high expression of PD-1 may constitute a mechanism by which PI3K signaling is orchestrated to differentially promote Tfh cell programs. These data corroborate earlier reports demonstrating that ligation of ICOS in the absence of TCR stimulation can still activate the PI3K pathway, which in this context helps explain the propensity towards Tfh differentiation in APDS patients ^8^. While these studies demonstrate that overactive PI3K signaling drives an increase in Tfh cells, these cells do retain some FOXO1 activity. FOXO1 is a transcription factor simultaneously required for sustained ICOS expression and an inhibitor of Tfh differentiation ^59^. Preite *et al*. went on to demonstrate that Pik3cd ^E1020K/+^ T cells are able to bypass the need for ICOS phosphorylation and inactivation of FOXO1, thereby promoting the Tfh gene expression program ^45^. Thus, study of APDS patients and mouse models of the disease has helped fill gaps in our understanding of how ICOS-mediated PI3K signaling promotes the Tfh and GC B cell programs.

## PIK3IP1/TrIP: a different kind of PI3K regulator

As discussed above, the PI3K pathway is subject to significant negative regulation at the level of PIP _3_, with several phosphatases acting to increase turnover of this second messenger. However, a distinct type of PI3K negative regulator appears to act at the level of PI3K catalytic activity itself. The initial report of PIK3IP1 described it as a novel kringle domain-containing protein ^60^. Interestingly, the same study identified a sequence in the cytoplasmic domain of PIK3IP1 with significant homology to the inter-SH2 domain of the p85 PI3K adaptor proteins. Indeed, this paper suggested that PIK3IP1 was capable of binding to a complex containing both p85 and p110 proteins. Finally, evidence was shown in this paper that expression of PIK3IP1 could inhibit the activity of PI3K. Although a precise mechanism was not identified, this important study suggested that PIK3IP1 might function to interfere with the allosteric activation of PI3K. A follow-up study by the same group reported that transgenic overexpression of PIK3IP1 in the liver could suppress the development of hepatocellular carcinoma in a mouse model ^61^, consistent with a pro-tumorigenic function of PI3K.

We became interested in the possible role of PIK3IP1 in lymphocytes as a result of the relatively high level of *PIK3IP1* mRNA in T and B cells of both mice and humans based on public gene expression databases. We confirmed the expression of PIK3IP1 on primary murine T cells and two established T cell lines ^25^. Particularly interesting is the expression of PIK3IP1 by Jurkat T cells, which are known to lack expression of two other PI3K negative regulators, PTEN and SHIP. We also showed that overexpression of PIK3IP1 could dampen PI3K signaling in response to anti-CD3/CD28 stimulation; conversely, siRNA-mediated silencing of endogenous PIK3IP1 led to enhanced T cell activation. These data revealed PIK3IP1 as a novel regulator of PI3K activity in lymphocytes and one uniquely positioned to act upstream of other known negative regulators of this pathway. Further studies revealed the importance of the extracellular kringle domain for PIK3IP1 inhibitory function ^26^. This finding raises the possibility that interaction with a ligand may modulate the ability of PIK3IP1 to control PI3K signaling in a context-dependent manner, although a specific ligand has yet to be identified.

A role for PIK3IP1 in T cell differentiation was demonstrated through *in vitro* helper T cell skewing experiments, where the absence of PIK3IP1 expression promoted a generally more inflammatory Th1 phenotype and was detrimental to the induction of Foxp3 ^+^ Tregs, which is consistent with work discussed above ^30^. These data were supported by an *in vivo* model of *Listeria monocytogenes* infection, where mice with T cell-specific knockout of PIK3IP1 showed a significantly lower bacterial burden compared to wild-type mice. Regulation of PIK3IP1 expression is only beginning to be understood, but recent evidence suggests that it is controlled in part by glutamine metabolism. Specifically, it was shown that glutaminolysis by T cells promotes a Th17 program, while restraining a Th1/CTL response, through direct effects on chromatin accessibility of multiple genes, including *Pik3ip1* ^62^. These results are consistent with our previous report that PIK3IP1 can restrain an inflammatory Th1 response ^26^. The biological impact of this PI3K regulator has recently been expanded to include control of anti-tumor immunity ^63^. This study employed mice with germline deficiency of PIK3IP1, which displayed significant resistance to the syngeneic transplantable tumors MC38 and B16F10 ^63^. However, the cellular and molecular mechanisms underlying this enhanced tumor resistance are not fully understood. As this review has briefly discussed, the PIK3IP1 regulation may have differential cell- and tissue-specific roles that warrant further investigation using more targeted disruption, e.g. in effector T cells vs. Tregs.

## Modulation of PI3K to improve efficacy of immunotherapy

Given the growing appreciation of how PI3K regulates lymphocyte cell fate decisions, there is emerging interest in targeting the PI3K pathway to improve cancer immunotherapy. While using PI3K inhibitors to directly inhibit the growth and survival of cancer cells is not a new concept ^64– 66^, the potential utility of combining such compounds with established immunotherapies to improve outcomes is more novel. One such approach has been the use of idelalisib (Zydelig®; Gilead), a PI3Kδ inhibitor approved for second- or third-line treatment of several types of B cell leukemia/lymphoma. It should be noted that the drug carries an FDA-mandated black box warning, owing mainly to the significant risk of systemic toxicity that can impact multiple organ systems ^67, 68^. As with many immunomodulatory drugs, the use of idelalisib also comes with a risk of opportunistic infection, which in this case may be due, at least in part, to a delay in CD8 ^+^ differentiation and pathogen clearance ^69^. This is consistent with murine studies showing that either knockout/inactivation of the PI3K catalytic subunit p110δ or the use of specific PI3K inhibitors (IC87114) led to impaired CD8 ^+^ T cell responses to both bacterial and viral infection ^70, 71^. In an attempt to circumvent these adverse events, Abu Eid *et al*. showed that inhibition of PI3Kδ *ex vivo*, prior to adoptive transfer of CD8 ^+^ T cells into tumor-bearing mice, led to a delay in the terminal differentiation of these cells and promoted their therapeutic activity ^69^. This is consistent with previous findings suggesting a role for PI3K in limiting the formation of memory precursor CD8 ^+^ T cells in response to acute infection with lymphocytic choriomeningitis virus (LCMV) ^72^. Thus, context-dependent PI3Kδ inhibition may allow for more precise therapeutic modulation of the immune response, with fewer side effects, than traditional systemic inhibition.

Of course, CD8 ^+^ T cells may not be the only relevant target of PI3K inhibition in settings of immunotherapy. As discussed above, precise regulation of PI3K activity is required for Treg function and homeostasis. Thus, targeted disruption of p110δ in Tregs has been shown to promote the regression of solid tumors in mice ^73^. In addition, more recent studies have shown that relatively preferential inhibition of Treg function or expansion by p110δ inhibition *in vivo* may promote the anti-tumor response to solid or liquid tumors ^40, 74– 77^. However, the interplay between PI3K inhibition and efficacy of other, now standard, immunotherapy agents (e.g. PD-1 blockade) remains unclear. As in any intervention that may modulate Treg function, tumor-specific approaches are desirable to avoid autoimmune sequelae as much as possible. Indeed, systemic inhibition of p110δ in Tregs has recently been associated with autoimmunity in mouse models ^78^ and has also been reported in clinical settings of PI3K use.

The inhibition of other members of the PI3K pathway, e.g. Akt and mTOR, has also been explored in settings of immunotherapy. Although partially inhibiting these kinases enhanced the acquisition of a memory T cell phenotype, this approach also limited T cell proliferation ^79^. This report is consistent with previous findings that pharmacological inhibitors of Akt ^80^ or of mTORC1 ^81^ enhance CD8 ^+^ memory precursor formation after LCMV infection. With the recent report of a role for BCAP in regulating effector versus memory differentiation of CD8 ^+^ T cells ^28^, it is possible that the key to improving CAR T cell therapy may be the identification of the right strategy to optimally “tune” PI3K signaling. As discussed above, the transmembrane protein PIK3IP1 represents a uniquely upstream regulator of the PI3K pathway whose impact on the effector/memory axis has yet to be fully understood. Despite the still-incomplete mechanistic understanding of PIK3IP1, its utility as a potential target for cancer immunotherapy has already shown promise ^63^.

While understanding the relationship between PI3K signaling and T cell biology can inform the development of new therapies, these same therapies can also help improve our understanding of T cell biology. Thus, in a follow-up study that examined the effects of the inhibition of PI3Kδ (via idelalisib), the Treg compartment was found to be uniquely sensitive to PI3Kδ inhibition when compared to other T cell types ^40^. Tregs treated with idelalisib displayed greatly reduced proliferation and signaling activity, even at the lowest concentrations of inhibitor employed. These data reinforce the model that some level of PI3Kδ activity is required for maintaining the Treg phenotype, whereas other types of T cells (e.g. effector T cells) are less sensitive to changes in this pathway. This relative resistance of effector T cells to PI3Kδ modulation may be explained through their ability to maintain PI3K signaling via the other (semi)redundant PI3K isoforms (PI3Kα/PI3Kβ), an idea that requires further exploration.

## Prospects for the future

Thirty-five years after the discovery of the first PI3K activity by Cantley and colleagues, we have learned a tremendous amount about the function and regulation of this lipid kinase family and its downstream effectors. Study of p110δ, which is relatively lymphocyte specific, and its role in the disease APDS has driven significant recent progress in the translation of this pathway to the clinic. Current and future studies should further drive the clinical application of PI3K pathway modulators in T and B cell responses, providing evidence that more specific inhibitors can be identified to fine-tune responses in ways that avoid excessive immunosuppression or toxicity. In this regard, further elucidation of how PI3K activity is regulated by more subtle modulators of the pathway like PIK3IP1 and BCAP may lead to novel approaches to tune PI3K activity for enhancement or inhibition of lymphocyte responses to infection or cancer.
